# Reply to the letter to the editor regarding: The impact of dietary acid load on superagers with exceptional cognitive abilities: A propensity score analysis of national health and nutrition examination survey (NHANES) 2011–2014

**DOI:** 10.1016/j.jnha.2024.100442

**Published:** 2024-12-10

**Authors:** Chen-Ying Lin, Hao-Hua An, Fan Wu, Jing-Na Lin

**Affiliations:** a Tianjin Union Medical Center, Tianjin Medical University, Tianjin 300121, China; b Department of Endocrinology, Health Management Center, Tianjin Union Medical Center, Nankai University Affiliated Hospital, Tianjin 300121, China; Department of Clinical Laboratory, Tianjin Union Medical Center, Nankai University Affiliated Hospital, Tianjin 300121, China; a Department of Endocrinology, Health Management Center, Tianjin Union Medical Center, Nankai University Affiliated Hospital, Tianjin 300121, China

Dear Editors-in-Chief,

We sincerely thank Storz et al. for their interest in our study titled, “The impact of dietary acid load on super-agers with exceptional cognitive abilities: a propensity score analysis of national health and nutrition examination survey (NHANES) 2011–2014” [1], as well as for their thoughtful comments and constructive questions. Their feedback provides a valuable opportunity to clarify our findings and address key aspects of our research in greater detail.

Super-agers are a rare subgroup of older adults aged 80 and above with exceptional cognitive abilities, defined by stringent criteria. Due to their low prevalence, most prior studies have relied on small, regional samples, limiting generalizability. Addressing this limitation, our study utilized the NHANES database – a large, nationally representative dataset – to systematically analyze the dietary patterns of super-agers. This approach reduces selection bias and enhances the reliability of findings. To overcome statistical challenges from small sample sizes, we employed propensity score analysis, well-suited for rare subgroups or uneven sample sizes, as it improves analytical efficiency, balances confounding factors, and reduces bias. Additionally, a sensitivity analysis involving 2522 participants provided valuable insights, complementing the limitations of focusing solely on super-agers.

The definitions of super-agers and typical-agers in our study were based on the criteria from the Northwestern University SuperAging Project [2]. Super-agers were defined as individuals aged 80 years or older with memory performance matching or exceeding the average of cognitively normal individuals aged 60–64, and non-memory cognitive scores at least within or above one standard deviation of the mean for their age group. Typical-agers were defined as cognitively healthy individuals aged 80 years or older whose cognitive scores fell within ±1.5 standard deviations of the mean for their age group but did not meet the super-ager criteria. Based on these definitions, individuals with severe cognitive impairment were excluded to ensure the study focused on older adults with preserved or exceptional cognitive abilities.

Storz et al. reported slightly positive values for potential renal acid load (PRAL) in individuals aged 80 and above, which differ from our findings. We believe this reflects a misinterpretation. Unlike Storz et al., who analyzed average PRAL values for the entire population aged 80 and above, our study focused on dietary pattern differences between super-agers and typical-agers. We found negative PRAL values for super-agers and 4.3 mEq/d for typical-agers, which aligns with or is slightly lower than Storz et al.’s data. These differences stem from our stringent inclusion criteria, which excluded individuals with severe cognitive impairments. Consequently, the super-ager and typical-ager groups represent older adults with better cognitive performance and potentially more favorable dietary patterns compared to the broader population aged 80 and above.

In addition to identifying significant differences in dietary acid load (DAL) indices between super-agers and typical-agers, our sensitivity analysis provided valuable additional evidence on the relationship between cognitive function and DAL indices. This analysis, which included 2522 participants aged 60 and above, revealed a significant decline in composite cognitive Z-scores with increasing PRAL values among individuals aged 70–79 and 80 and above. This trend, shown in Figure 3 of the main text [1] and Supplementary Fig. 1, further supports the negative correlation between PRAL and cognitive function, offering clearer and more robust evidence of the impact of DAL on cognitive performance.

Storz et al. reported that up to 20% of their sample had insufficient protein intake, consistent with our preliminary data review. We believe this data accurately reflects the study population's nutritional intake, as no extreme outliers or estimation errors were identified. Therefore, we retained these samples rather than excluding individuals with low protein intake. Removing such samples could skew results away from real-world data and lose important information, especially as protein intake is critical for calculating PRAL. Exclusion could alter DAL value distributions, reduce analytical sensitivity, and underestimate the impact of low protein intake on cognition. To ensure comprehensive and representative findings, we used the complete dataset, which aligns better with real-world conditions and provides more reliable evidence on the role of dietary acid load in cognitive health.

In this study, DAL indices, including PRAL, were calculated based on non-bicarbonate anions (protein and phosphorus) and mineral cations (potassium, magnesium, and calcium) to assess dietary acid load. As shown in Supplementary Table 1, super-agers had slightly lower protein and phosphorus intake but higher potassium, calcium, and magnesium intake compared to typical-agers, indicating that differences in PRAL and other DAL indices result from the combined effects of multiple nutrients rather than protein alone. Additionally, typical-agers had significantly lower potassium, calcium, and magnesium intake than younger-agers (*P* < 0.05), while super-agers showed similar levels to younger-agers (*P* > 0.05). These findings suggest that super-agers' dietary patterns, particularly mineral cation intake, align more closely with those of younger individuals with good cognitive function. PRAL, by integrating multiple nutrient differences, offers a comprehensive reflection of dietary acid load's impact on cognitive function. These findings provide a broader perspective on the relationship between dietary acid load and cognitive performance and address some of the concerns raised by Storz et al. regarding dietary parameters. We appreciate their interest and the opportunity to clarify these critical points.

## Declaration of competing interest

The authors declare that they have no known competing financial interests or personal relationships that could have appeared to influence the work reported in this paper.

## References

[1] Lin C.Y., Li F., An H.H., Zhai Y.J., Li J.B., Qiu H.N. (2024). The impact of dietary acid load on super-agers with exceptional cognitive abilities: a propensity score analysis of national health and nutrition examination survey (NHANES) 2011-2014. J Nutr Health Aging.

[2] Harrison T.M., Weintraub S., Mesulam M.M., Rogalski E. (2012). Superior memory and higher cortical volumes in unusually successful cognitive aging. J Int Neuropsychol Soc.

