# Supplementary material for: Reply to the letter to the editor regarding: The impact of dietary acid load on superagers with exceptional cognitive abilities: A propensity score analysis of national health and nutrition examination survey (NHANES) 2011–2014
Source: J Nutr Health Aging. 2024 Dec 10;29(2):100442. doi: 10.1016/j.jnha.2024.100442 (PMC12180057; doi:10.1016/j.jnha.2024.100442)
Supplement: Supplementary file 1 [file mmc1.docx]

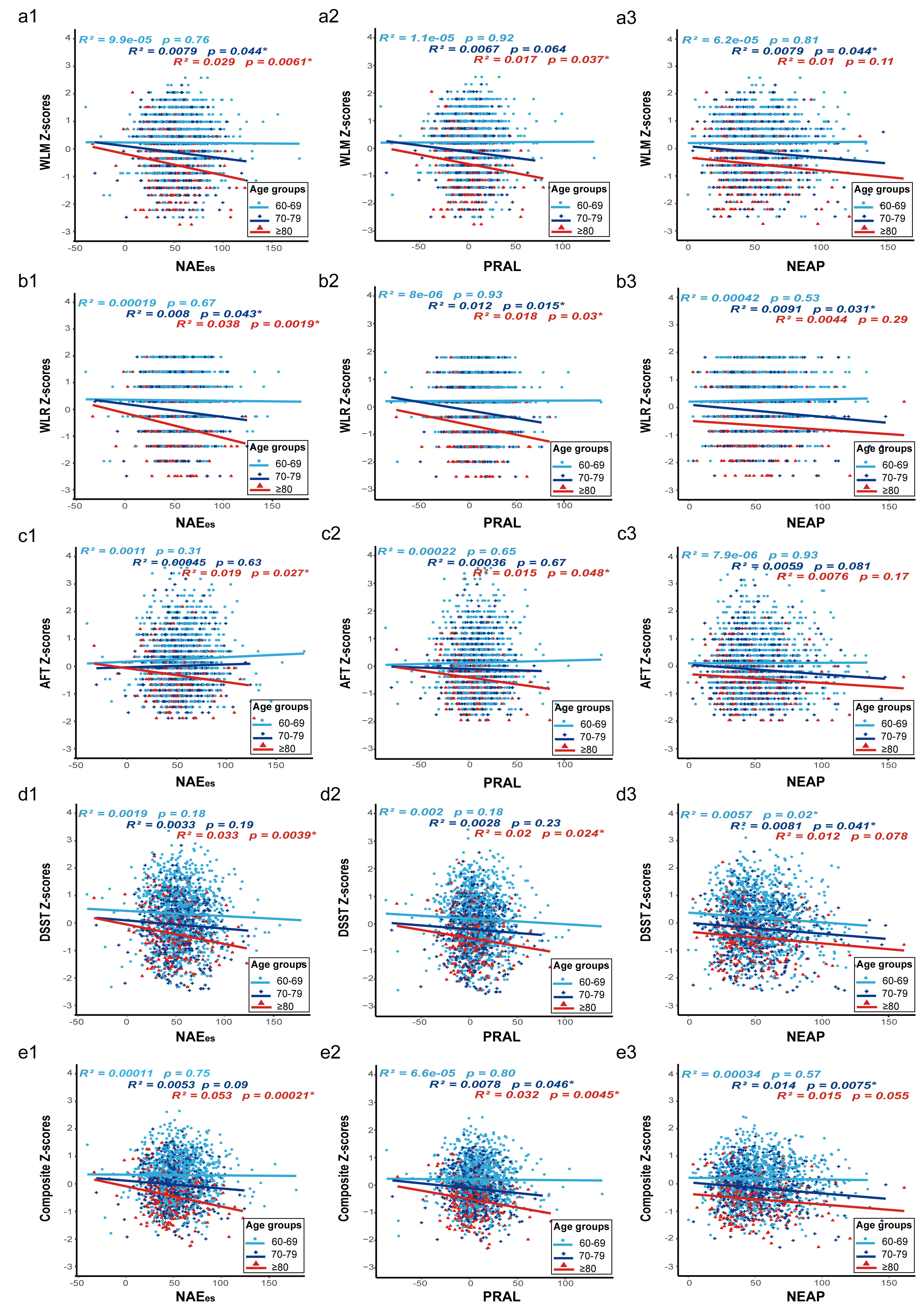
**Supplementary Figure 1** Age-stratified Pearson correlation analysis between dietary acid load (DAL) indices and cognitive function. Correlations are shown for **(a1-a3)** standardized Z-scores of word list memory (WLM), **(b1-b3)** word list recall (WLR), **(c1-c3)** animal fluency test (AFT), **(d1-d3)** digit symbol substitution test (DSST), and **(e1-e3)** overall cognitive function composite Z-scores with DAL-related indices (NAE_es_, PRAL, and NEAP). Each subfigure is stratified by age categories: 60-69, 70-79, and 80+. Abbreviations: NAE_es_, estimated net acid excretion; PRAL, potential renal acid load; NEAP, net endogenous acid production. **P*-value < 0.05.

**Supplementary Table 1** Comparison of dietary acid load indices and nutrient intake among Super-Agers, Typical-Agers, and Young-Agers.

| Characteristic | Super-Agers  N = 33 | Typical-Agers  N = 221 | *P-*Value * | Young-Agers  N = 731 | *P* *-*Value **^a^** | *P-*Value **^b^** |
| --- | --- | --- | --- | --- | --- | --- |
| Age | ≥80 years | ≥80 years | *-* | 60-69 years | *-* | *-* |
| NAE_es_ (mEq/d) | 35.5 (18.8) | 47.5 (21.6) | 0.001 | 54.5 (24.6) | <0.001 | <0.001 |
| PRAL (mEq/d) | -4.4 (17.9) | 4.3 (19.5) | 0.009 | 9.2 (23.0) | <0.001 | 0.008 |
| NEAP (mEq/d) | 38.7 (18.7) | 47.6 (19.7) | 0.012 | 51.7 (20.9) | <0.001 | 0.050 |
| Protein (gm/d)） | 57 (25) | 65 (31) | 0.111 | 80 (36) | <0.001 | <0.001 |
| Phosphorous (mg/d) | 1,103 (479) | 1,144 (499) | 0.678 | 1,369 (615) | 0.007 | <0.001 |
| Potassium (mg/d) | 2,603 (905) | 2,496 (1,088) | 0.524 | 2,851 (1,190) | 0.283 | <0.001 |
| Calcium (mg/d) | 867 (447) | 819 (443) | 0.623 | 949 (569) | 0.715 | 0.004 |
| Magnesium (mg/d) | 284 (102) | 264 (117) | 0.305 | 317 (155) | 0.120 | <0.001 |

NAEes, estimated net acid excretion; PRAL, potential renal acid load; NEAP, net endogenous acid production. Continuous variables analyzed using the t-test tailored for complex survey samples. P-values for comparisons: *P < 0.05 for super-agers vs. typical-agers. ^a^ P < 0.05 for super-agers vs. young-agers. ^b^ P < 0.05 for typical-agers vs. young-agers.
